# A vicissicaudatan arthropod from the Silurian Herefordshire Lagerstätte, UK

**DOI:** 10.1098/rsos.230661

**Published:** 2023-08-02

**Authors:** Derek E. G. Briggs, David J. Siveter, Derek J. Siveter, Mark D. Sutton, David Legg, James C. Lamsdell

**Affiliations:** ^1^ Department of Earth and Planetary Sciences, and Yale Peabody Museum, Yale University, New Haven, CT 06520-8109, USA; ^2^ School of Geography, Geology and the Environment, University of Leicester, Leicester LE1 7RH, UK; ^3^ Earth Collections, University Museum of Natural History, Oxford OX1 3PW, UK; ^4^ Department of Earth Sciences, University of Oxford, Oxford OX1 3AN, UK; ^5^ Department of Earth Sciences and Engineering, Imperial College London, London SW7 2BP, UK; ^6^ Department of Earth and Environmental Sciences, University of Manchester, Manchester M13 9PL, UK; ^7^ Department of Geology and Geography, West Virginia University, 98 Beechurst Avenue, Brooks Hall, Morgantown, WV 26506, USA

**Keywords:** Herefordshire Konservat-Lagerstätte, Silurian, Arthropoda, Vicissicaudata, Nekton

## Abstract

A new arthropod, *Carimersa neptuni* gen. et sp. nov., is described from the Silurian (Wenlock Series) Herefordshire Konservat-Lagerstätte, UK. The head bears pedunculate eyes and five pairs of appendages. Triflagellate antennae are followed by two pairs of uniramous limbs each with an endopod bearing a pronounced gnathobasic basipod. The posterior two pairs of head limbs and all trunk limbs bear an endopod, exopod and filamentous exite. The trunk consists of 10 appendage-bearing segments followed by an apodous abdomen of four segments. The arthropod resolves as sister taxon to *Kodymirus* and *Eozetetes* + Aglaspidida. It is the first representative of Vicissicaudata reported from the Herefordshire Lagerstätte and the first Silurian example with well-preserved appendages. The preservation of a cluster of radiolarians apparently captured by the trunk appendages is the first direct association of predator and prey discovered in the Herefordshire fauna, and suggests that *Carimersa* was a nektobenthic form that used its gnathobasic basipods in microdurophagy.

## 1. Introduction

The mid-Silurian (approx. 430 Myr BP) Herefordshire Konservat-Lagerstätte preserves the most diverse assemblage of soft-bodied fossils known from the Silurian. The fossils are preserved in calcareous concretions in a volcanic ash [1]. The taxa provide important range extensions and many of them are significant to our understanding of the evolutionary history of the groups to which they belong. Arthropods make up approximately 45% of identified specimens. Seventeen new taxa have been described including representatives of lobopodians, megacheirans, pycnogonids, chelicerates, marrellomorphs and crustaceans [2]. Early Palaeozoic arthropods continue to attract attention as Konservat Lagerstätten yield a steady stream of new taxa, most without a biomineralized exoskeleton. New discoveries contribute morphologies that are important in revealing relationships within Arthropoda and clarifying the early evolution of the group. Exceptional preservation is rarer after the Cambrian so Ordovician and Silurian examples are significant in determining the later diversification and longevity of clades. Here we describe a new exceptionally preserved vicissicaudatan, *Carimersa neptuni* gen. et sp. nov., the first from the Herefordshire deposit, and analyse its phylogenetic position and ecology.

## 2. Material and methods

**Figure 1.**
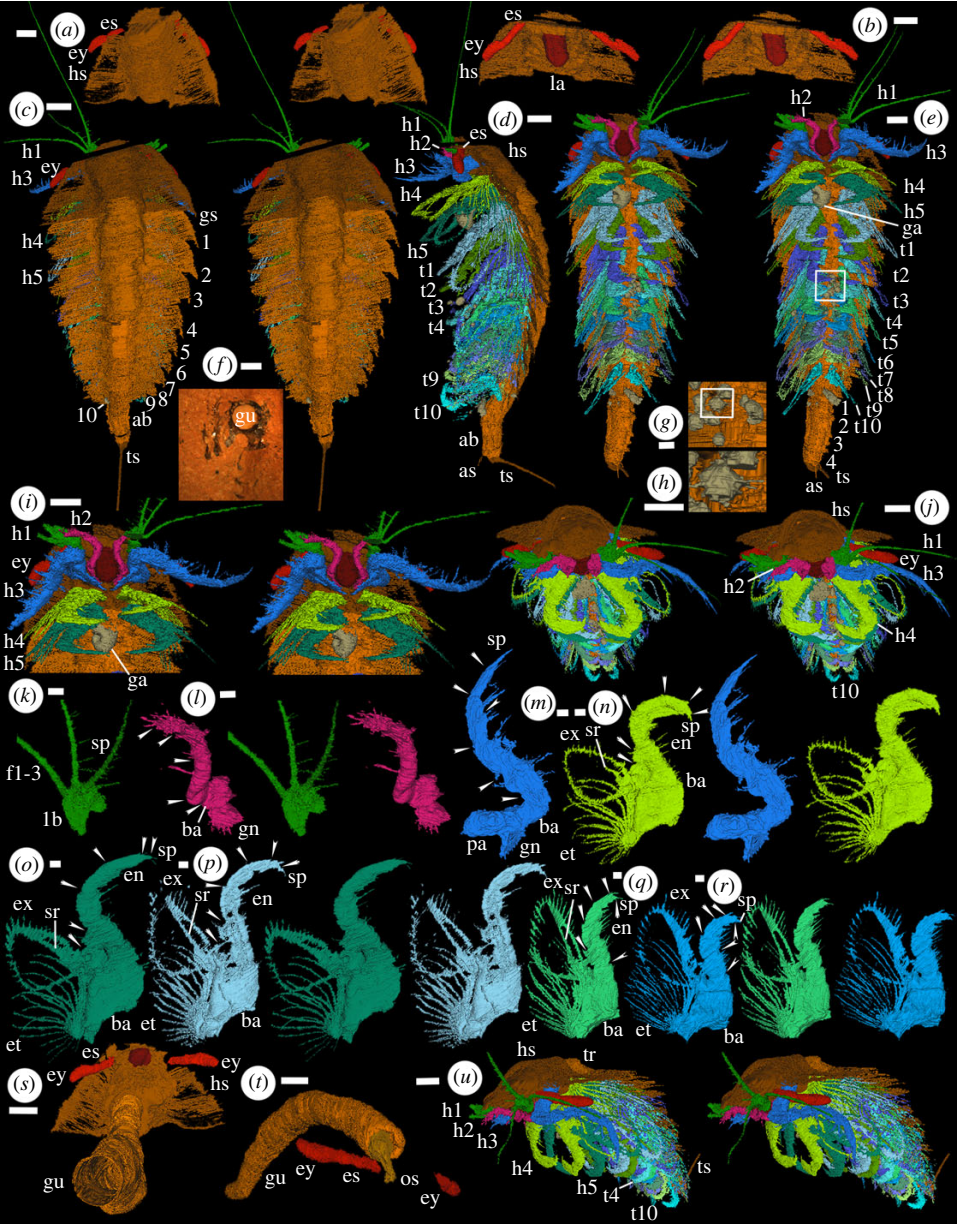
Holotype of *Carimersa neptuni*, exoskeleton and soft parts (OUMNH PAL-C.376503): (*a–e*, *i–u*) ‘virtual' reconstructions (*a–c*,*e*,*i–r*,*u* are stereo-pairs); (*f*) specimen in rock. The exact boundary between structures such as body and limbs, as indicated by colour changes, involves some interpretation. (*a*) Head shield, dorsal view showing eyes. (*b*) Head shield, ventral oblique view showing eyes and hypostome/labrum complex, other soft parts omitted. (*c*) Dorsal view. (*d*) Left lateral view. (*e*) Ventral view. (*f*) Section through abdomen showing sediment filled body cavity and other features. (*g*,*h*) Cluster of radiolarians and a single specimen between trunk appendages 2–5. (*i*) Head in ventral view. (*j*) Anterior oblique view. (*k*) Left appendage 1, dorsal view of proximal part. (*l,n–r*) posterior oblique view of right head appendages 2 (*l*), 4 (*n*), 5 (*o*), trunk appendages 1 (*p*), 5 (*q*), 6 (*r*). (*m*) Left appendage 3, anterior oblique view. (*s*) Posteroventral view of the gut, head shield, eyes and hypostome/labrum complex; all other features omitted. (*t*) Right lateral oblique view of gut (showing posteriorly flexed oesophagus) and eyes; all other features omitted. (*u*) Left anterior oblique view. ab, abdomen; as, abdominal spine; ba, basipod; es, eye stalk; ey, eye; et, exite; ex, exopod; f1–3, flagella; ga, gastropod; gn, gnathobase; gs, genal spine; h1–h5, head appendages; gu, gut (or possibly body cavity); hs, head shield; lb, limb base; la, hypostome/labrum complex; os, oesophagus; pa, precoxal area; sp, spine(s); sr, strengthening rod; t1–t10, trunk appendages; tr, trunk; ts, tail spine. Numbers refer to trunk tergites (1–10) and abdominal segments (1–4). Arrows in (*l–r*) indicate podomere boundaries. Scale bars: *a–f*, *i*, *j*, *o–u* are 2 mm; *g*, *h*, *k–n* are 0.5 mm.

A single specimen of the new arthropod is known. The concretion was serially ground at intervals of 30 µm except for the portions containing the antennae and tail spine which were ground at 20 µm. The surfaces were submerged under a thin layer of water and photographed with a Leica DFC420 digital camera mounted on a Leica MZ8 binocular microscope. The SPIERS software suite was used to remove extraneous material from the images and to reconstruct the arthropod (figure 1) as a three-dimensional ‘virtual fossil' [3–5]. Datasets from the serial grinding, together with the final three-dimensional model in VAXML/STL format, are held by the Oxford University Museum of Natural History (OUMNH), and are also available from the Dryad Digital Repository (https://doi.org/10.5061/dryad.hmgqnk9ds).

The new arthropod was coded into a novel dataset (electronic supplementary material, based largely on [6]), to explore its relationships among vicissicaudatan arthropods. This new dataset consists of 104 characters coded for 78 taxa. It was analysed under both maximum-parsimony and Bayesian optimality criteria using TNT v. 1.5 [7] and MrBayes 3.2.7a [8] respectively.

The parsimony search strategy employed 10 000 random addition sequences with all characters unordered and of equal weight [9], each followed by tree bisection-reconnection (TBR) branch swapping. Jackknife [10], Bootstrap [11] and Bremer [12] support values were also calculated in TNT. Bootstrapping was performed with 50% resampling for 10 000 repetitions, while jackknifing was performed using simple addition sequence and tree bisection-reconnection branch swapping for 10 000 repetitions with 33% character deletion.

The Bayesian analysis comprised four independent runs of 100 000 000 generations and four chains, each under the maximum-likelihood model for discrete morphological character data with gamma-distributed rate variation among sites (Mkv + Γ [13]). Trees were sampled every 1000 generations, resulting in 1 000 000 trees per run, with the first 25 000 sampled trees (25 000 000 generations) of each run discarded as burn-in. The 50% majority rule consensus tree was calculated from the remaining 750 000 sampled trees across all four runs [14]. The frequency at which a clade occurred among the sampled trees included in the consensus tree was used to calculate posterior probabilities.

The parsimony analysis resulted in 26 most parsimonious trees, the strict consensus of which is broadly congruent with that derived from the Bayesian analysis, including the position of *Carimersa* (electronic supplementary material, figures S1, S2).

## 3. Systematic palaeontology

Phylum Arthropoda [15]

Artiopoda [16]

Vicissicaudata [17]

**Emended diagnosis** (after [18])

Artiopods with trunk composed of 6 to 19 articulated segments (generally 10 to 12) all, except for the posteriormost one (exceptionally up to four of the posteriormost), with wide (tr.) pleurae; last trunk segment bearing a pair of non-walking appendages, sometimes secondarily lost.

Genus *Carimersa* gen. nov.

Type and only species *Carimersa neptuni* sp. nov.

### 3.1. Etymology

Latin, *caris* (shrimp, crab) + *mersa* (buried), referring to entombment in volcanic ash (gender feminine); *neptuni* after the Roman god of the sea who carried a trident, alluding to the triflagellate antennae.

### 3.2. Diagnosis of genus and species

Head bearing five paired appendages, the first bearing three flagella, the second and third uniramous with a substantial gnathobasic basipod; the posterior two head appendages and all trunk appendages bear a robust spinous endopod, a flap-like exopod with a strengthening rod, and an exite proximally with an array of long filaments. The trunk consists of 10 appendage-bearing segments, an apodous abdomen of four segments and a tail spine.

### 3.3. Material

Only the holotype, OUMNH PAL-C.376503, a specimen with soft parts reconstructed in three dimensions (figure 1*a–f*, *i–u*).

### 3.4. Locality and horizon

Herefordshire, England, UK; Wenlock Series, Silurian.

### 3.5. Description

The body consists of a cephalon, thorax and a short apodous abdomen which terminates in a single long tail spine (figure 1*c–e*,*j*,*u*). The body is preserved as a thin-walled sediment-filled cavity; apart from part of the gut no internal organs are preserved. The body measures about 30 mm along its length from the anterior margin of the cephalon to the posterior extremity of the trunk (excluding the tail spine). The maximum width, which occurs at the rear of the cephalon, is about 12.5 mm. The tail spine is incompletely preserved (figure 1*c–e*): its distal termination was outside the concretion and about 5.5 mm remains. The cephalon bears five pairs of appendages (figure 1*i*). The thorax consists of 10 appendage-bearing segments (figure 1*d,e*).

The head shield is semicircular in outline extending posterolaterally into short triangular (genal) spines (figure 1*a*,*c*). It overlaps the anterior portion of the first trunk tergite. The axis of the head shield is moderately raised and expands anteriorly by about 30% to a rounded frontal margin (figure 1*a*,*c*). Ecdysial sutures are not evident but might not have been revealed by serial grinding. The raised axis continues into the trunk where each tergite extends posterolaterally into a spine similar to the genal spines (figure 1*c*).

The eyes are pedunculate, the stalk inserting anterior of appendage 1 as revealed by the complete reconstruction of the attachment of the right eye (figure 1*i*,*j*,*u*). The stalks are broad, sub-circular in cross section with a diameter of approximately 0.5 mm, lack evidence of segmentation (figure 1*b*) and extend beyond the margin of the head shield (figure 1*a*,*j*). The eyes are elongate, slightly dorsoventrally compressed, and a little broader than the stalk. There is a slight constriction where the eye is attached; there is no evidence to indicate whether this represents an articulation (figure 1*b*).

All appendages except the first three head appendages, which are clearly differentiated from the rest, are biramous. Appendages 4 and 5 are similar to the trunk appendages but lie beneath the head shield (figure 1*e*,*i*). Head appendage 1 inserts level with the anterior margin of the hypostome/labrum complex (figure 1*i*). It consists of a limb base of at least two podomeres and bears three flagella (figure 1*k*). The limb base of the right appendage and its attachment to the head are reconstructed but only the bases of the flagella on this appendage are available (figure 1*c*,*e*,*i*). There is a hiatus through the base of the left limb but the flagella on that limb can be reconstructed (figure 1*c*). The longest flagellum (the most dorsally positioned and directed) is attached to the distal extremity of the limb base. The other two flagella appear to be attached just proximal to this but to the same distalmost part of the limb base (figure 1*k*). The relationship between the spines preserved in the proximal part of the flagella and podomeres along its length is not clear. If the position of the spines corresponds to podomeres extrapolation suggests that there may be at least 50. Only the middle (in dorsal view) of the three flagella was recovered to its termination.

Head appendage 2 (only the right recovered to its termination) flanks the hypostome/labrum complex, inserting near its rear margin (figure 1*i*). The large limb base (representing at least a basipod) has a well-developed gnathobase, with approximately seven or eight adaxially projecting spines apparently arranged in an inner and outer row along its adaxial margin (figure 1*l*). The gnathobase is demarcated from the rest of the basipod by a groove which runs normal to the attachment of the appendage to the trunk and may represent an articulation (figure 1*l*). The spinose ramus (presumed endopod), which projects anteriorly from the basipod and flexes abaxially, comprises at least four podomeres. The relationship between the spines along its length and possible podomere boundaries is not clear. The terminal podomere bears many short spines including two–three distal ones which project forward (figure 1*l*).

Head appendage 3 is similar to the second but larger (figure 1*i*,*j*,*m*). It bears a large flattened gnathobasic basipod with a single vertical row of approximately 10 spines that project adaxially and somewhat posteriorly towards the mouth opening; the most ventral of these spines (evident on the right limb) is the largest. The more proximal, precoxal part of the limb base is stout, dorsally projecting, inclined slightly outward, and attached to the head on its proximal margin (figure 1*m*). It is separated from the gnathobasic basipod by a constriction and groove which presumably represent an articulation. Distal to the basipod the endopod consists of at least five podomeres and one large and two small terminal spines. There are a few spines on the outer margin of some of the podomeres closest to the gnathobasic basipod and many spines, seemingly arranged in pairs, along the inner margin at the mid-length of the podomeres. The distalmost podomere, immediately proximal to a terminal spine, bears a pair of ventral spines distally.

Appendages 4 and 5 lie beneath the head shield and appear to attach to the head (figure 1*i*). The basipod of appendage 4 (figure 1*n*) is approximately triangular in shape and much broader than that of appendage 3. The proximal part of the basipod is not obviously spinose and is separated from the basipod of the opposing member of the pair by a narrow gap (figure 1*e*). An exite bearing an array of curved filaments is attached proximally on the outer margin of the basipod. An oval flap-like exopod is attached distally to the basipod (figure 1*n*). The endopod is robust; the podomeres are difficult to enumerate due to the orientation of the slices but there appear to be five, plus small terminal spines, with a marked geniculation between podomeres 2 and 3, which are the longest (figure 1*n*). There are short spines, several arranged in pairs, along the inner margin of the first three podomeres and a few short spines on the outer margin. The thickened margin of the exopod is fringed by short slender spines or setae. A linear structure, presumably a strengthening rod, incompletely preserved in appendage 4 (figure 1*n*), separates a narrow inner portion of the exopod from a wider outer portion. The exite consists of an array of nine pairs of more robust long filaments that radiate from their attachment to the basipod, the opposing members of each pair curving toward each other (figure 1*n*). Head appendage 4, and those following it, are strongly compressed antero-posteriorly, and the podomeres of the endopod become increasingly so distally. This is not an artefact of collapse or flattening as it is a feature of all the limbs in the series, even though they are preserved in a range of different orientations. Head appendage 5 (figure 1*o*) is very similar to the fourth. The strengthening rod in the exopod, which separates a narrow triangular area along the inner margin (facing the endopod) from a wider outer area, is more completely preserved than in head appendage 4.

The hypostome/labrum complex (*sensu* [19]) is a small posteroventrally directed projection with parallel sides, a dorsal surface that is convex posteriorly and a flat ventral surface inclined at about 30° to the body wall (figure 1*b*,*e*,*s*). It terminates posteriorly in a blunt cone (figure 1*b*,*i*). The hypostome/labrum complex is flanked laterally and posteriorly by the gnathobasic basipods of head appendages 2 and 3 respectively (figure 1*i*,*j*). The anteriormost part of the gut trace (oesophagus) narrows and bends posteroventrally to the mouth opening which presumably lies dorsal of the hypostome/labrum complex and faces rearward (figure 1*t*). The rest of the trunk is preserved as a thin-walled hollow tube which may represent the body cavity rather than the gut trace (figure 1*f*,*s*).

Trunk appendage 1 (figure 1*p*) is the first of 10 in the thorax. They are similar morphologically to head appendage 5 (figure 1*d*,*e*,*u*). Trunk appendage 2 appears to show a much narrower exopod but only the outline of the inner portion (a narrow triangle) is evident: the outer margin is too poorly preserved to be detected in our reconstruction. Trunk appendage 3 reveals that the morphology of the exopod is the same or similar to those of more anterior appendages. Trunk appendage 4, like right trunk appendage 3, is traversed by the cut between the two parts of the concretion, trunk appendage 5 less so. The morphology of the posterior appendages is the same as that of the rest of the series (figure 1*d*,*e*,*q*,*r*). The exopod on the last limb, trunk appendage 10, is poorly preserved, particularly on the right side: the exite of this limb is barely evident, even in raw tomograms, but this probably reflects poor preservation rather than absence. The trunk appendages show a gradual reduction in size such that trunk appendage 10 is about half the size of trunk appendage 1 (figure 1*d*,*e*,*u*).

The 10 tergites of the thorax overlap posteriorly (figure 1*c*,*d*). The pleura are very thin and more completely preserved on the right side; they project from the raised axis with slight ventral curvature and extend a similar distance laterally to the appendages beneath them. The pleura of the first seven tergites (those corresponding to trunk appendages 1 to 7) terminate in a posteriorly directed point; the following two pleura are more laterally directed with a blunt termination (figure 1*c*). The tergites extend a much shorter distance laterally toward the rear of the trunk. The pleura corresponding to trunk appendage 10, the last one, are very reduced. The pleura are thickened slightly along their anterolateral margin.

A narrow extension of the trunk beyond the last trunk appendage, here interpreted as the abdomen, is approximately the same width as the trunk axis and lacks appendages (figure 1*c-e*). The segments appear to be tubular with no dorsoventral separation. Segment boundaries are difficult to distinguish but we identify four anterior to the tail spine. The abdomen is flexed dorsally at about 30°, and slightly laterally, at about 1/3 its length (figure 1*c*,*d*). Two short spines project from the posteroventral margin of the left side of the segment immediately preceding the tail spine, and they were presumably also present on the right side (figure 1*e*). The anus is represented by a large circular aperture that leads to the body cavity (figure 1*f*,*s*). It underlies the triangular base of a long tail spine which was presumably movable and projects dorsally at about 75° to the abdomen (figure 1*d*). The spine curves slightly ventrally and maintains a near constant width, but its full length was not captured by our reconstruction.

A gastropod lies in the midline between the last head appendage 5 and trunk appendage 1 (figure 1*e*,*i*,*j*). A concentration of much smaller spherical objects, about 15 in number, is preserved within the axial space enclosed by trunk appendages 2 to 5 (figure 1*d*,*e*,*j*). Although they vary in shape and size as reconstructed, one shows four short radially projecting spines, roughly equidistant in spacing, in the equatorial plane (figure 1*g*,*h*) indicating that they represent radiolarians (which occur in other concretions from the locality [20]). The exception is a small gastropod near the base of left trunk appendage 5.

## 4. Discussion

### 4.1. Affinities and evolutionary significance

*Carimersa* shares features with a diversity of arthropods. The head bears pedunculate eyes and five appendages, the first three differentiated, the last two similar to those of the trunk. Such a similarity between posterior trunk appendages is known in other arthropods, including living crustaceans such as cephalocarids, and *Tanazios* [21] and *Cascolus* [22] from the Herefordshire Lagerstätte. The three long flagella of the first head appendage resemble those in stomatopod malacostracans, in the Cambrian stem mandibulate *Oelandocaris* [23], and in *Enalikter* [24] and *Cascolus* [22] from the Herefordshire Lagerstätte. A dorsolateral extension of the basipod of the third head appendage is similar to that in the Burgess Shale arthropod *Sidneyia* and in living horseshoe crabs [25,26].

The trunk limbs of *Carimersa*, as well as the two pairs at the rear of the head, comprise a basipod which attaches to the body, a segmented endopod, a flap-like exopod divided by a rod and a filamentous exite. Similar filamentous structures are present on the proximal part of the exopod in the megacheiran *Bundenbachiellus* from the Lower Devonian Hunsrück Slate of Germany [27]. The exopod and exite of *Carimersa* combined are reminiscent of the tripartite exopod in the limbs of the Cambrian artiopod *Emeraldella brocki* [28]. The filaments of the exite of *Carimersa* are similar to the lamellae on the proximal part of the *Emeraldella* exopod which likewise articulates with the basipod. The divided exopod of *Carimersa* with its fringe of setae is similar to the middle and flap-like distal part of the *Emeraldella* exopod. It is clear, however, that the exite and exopod of *Carimersa* are not contiguous and attach to the basipod separately, although this arrangement could be derived from the exopod of *Emeraldella*. The apodous abdomen consists of four segments and terminates in a long tail spine like that in Cambrian taxa such as *Habelia* [29,30], *Emeraldella* [28,31] and *Tanglangia* [32] and in living horseshoe crabs.

**Figure 2.**
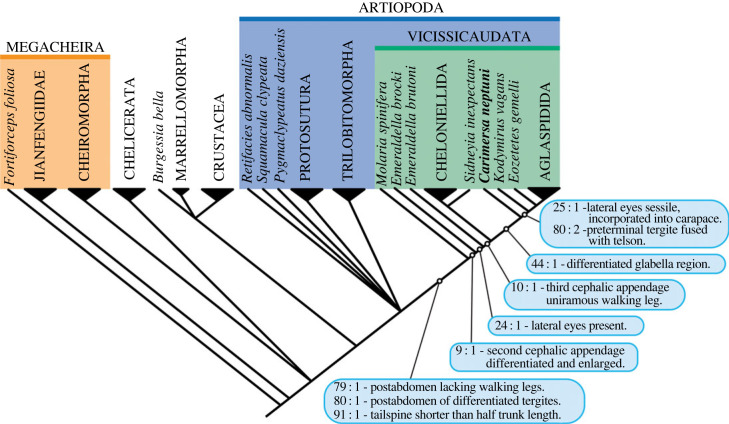
The phylogenetic position of *Carimersa neptuni* among Vicissicaudata based on parsimony and Bayesian analyses (electronic supplementary material, figures S1, S2). Key synapomorphies are indicated.

The unique combination of morphological characters supports the assignment of *Carimersa neptuni* to a new genus and species. Our phylogenetic analysis (figure 2; electronic supplementary material, figure S1) resolves it within Artiopoda. The Herefordshire Lagerstätte has already yielded trilobites [2,33], and chelicerates in the form of *Offacolus* [34] and *Dibasterium* [35] but *Carimersa* is the first artiopod outside these two major clades to be discovered in this Lagerstätte. *Carimersa* falls out as sister taxon to *Kodymirus* and *Eozetetes* + Aglaspidida. *Carimersa* is the first representative of the larger clade Vicissicaudata [17,36], which includes Aglaspidida and Cheloniellida, from the Herefordshire Lagerstätte and the first Silurian example with well-preserved appendages. The youngest representative of Aglaspidida is *Chlupacaris dubia* from the Upper Ordovician of Morocco [37]. Cheloniellida, in contrast, ranges to the Lower Devonian where it is represented by *Cheloniellon* from the Hunsrück Slate of Germany [38] and *Paraduslia* from Russia [39]. While we acknowledge that there is only a single well-preserved specimen, the diagnosis of Vicissicaudata is here emended (after [18]): the inclusion of *Carimersa* requires only that the abdomen exceptionally includes up to four, as opposed to up to ‘two/three', segments.

The similarities between *Carimersa* and *Emeraldella* prompt a discussion of Xenopoda which was erected 100 years ago [40] as a subclass for the Burgess Shale arthropods *Sidneyia* and *Emeraldella*, taxa with ‘one pair of uniramous antennae, biramous appendages on anterior part of trunk, modified endopodites on cephalon'. Several phylogenetic analyses have recovered these genera as a clade [41–44]. Ortega-Hernández *et al*. ([17]; see also [45,46]), in contrast, recovered a paraphyletic Xenopoda with *Sidneyia* and *Emeraldella* leading to Cheloniellida. Ortega-Hernández *et al*. [17] established Vicissicaudata to include Xenopoda, Cheloniellida and Aglaspidida and discussed older papers dealing with the relationships of these taxa ([17], p. 38). Legg *et al*. [36] likewise recovered a paraphyletic Xenopoda leading to Cheloniellida. Edgecombe *et al*. [47] recovered the same topology using the matrix in [7] but obtained a different solution using the matrix in [48]. They pointed out that the result is ‘sensitive to differences in taxonomic and character sampling' [47, p. 94]. Aria & Caron [44] recovered *Sidneyia* and *Emeraldella* as a clade but as sister to xandarellids and trilobites. Lerosey-Aubril *et al*. [18] retrieved a paraphyletic Xenopoda, with *Sidneyia* and *Emeraldella* on the branch leading to Aglaspidida but separated by Cheloniellida. Our results, like most previous phylogenies, find no support for Xenopoda as a clade.

### 4.2. Mode of life

*Carimersa* is the second Herefordshire arthropod, following the parasitic relationship of the pentastomid *Invavita* on the ostracod *Nymphatelina* [49], to preserve evidence of a direct association between taxa. The presence of shelly microfossils, including radiolarians and gastropods, in the midline of the trunk between the appendages of *Carimersa* has not been observed in any other Herefordshire arthropod and is unlikely to be the result of chance. Radiolarians are not common in the Herefordshire concretions with recovery rates of 2–25 tests per 500 g sample [20]. The Herefordshire radiolarian fauna has affinities with assemblages in the Urals, the Canadian Arctic and Alaska [20], which is consistent with dispersal of planktic forms around the margins of Laurentia.

The flattened nature of the trunk appendages and the posterior two head appendages of *Carimersa* signal a swimming mode of life. The morphology of the appendages indicates that it was unlikely to be a filter or deposit feeder. The first head appendage consists of three antenna-like rami which were presumably sensory in function. The second and third head appendages were equipped with pronounced gnathobasic basipods that project toward the rear of the hypostome/labrum complex and presumably processed and manipulated food in the immediate vicinity of the mouth. The robust proximal part of head appendage 3 of *Carimersa* resulted in a strong attachment to the head. The basipod of the fourth head appendage and succeeding appendages is not obviously spinose, but a narrow gap between the opposing limb bases probably facilitated the transport of food items to the mouth, although there is no food groove. The inward facing spines on the endopods may have assisted in capturing prey and moving it forward. The sediment content of the gut may have been introduced during burial; other Herefordshire arthropods have sediment-filled guts although their appendages are incompatible with deposit feeding [21,50].

Radiolarians are preyed upon today by nektic and planktic crustaceans such as copepods, euphausids, and some penaeids and sergestids [51]. *Carimersa* is an order of magnitude larger than copepods but in the range of the other crustacean taxa. It is equipped with gnathobasic basipods which could have functioned in microdurophagy. Benthic radiolarians are unknown, however, and radiolarian tests on the sediment surface would have lost their soft parts through decay and lacked any nutritional value. The anterior of the two gastropods between the limbs is larger than the norm for planktonic larvae [52]. Thus *Carimersa* may have fed on and above the sediment surface in a nektobenthic lifestyle similar to that inferred for many of the other Herefordshire arthropods [2].

## Data Availability

The raw serial-grinding data and triangle-mesh model of holotype specimen OUMNH PAL-C.376503 (in VAXML/STL format) are available from the Dryad Digital Repository: https://doi.org/10.5061/dryad.hmgqnk9ds [53]. Cladistic character-matrix datasets supporting this article have been uploaded as part of the electronic supplementary material [54].
